# Mortality among hospitalised patients with schizophrenia : Data from a Tunisian psychiatric hospital

**DOI:** 10.1192/j.eurpsy.2026.11283

**Published:** 2026-07-31

**Authors:** G. Ltaief, A. Karmous, A. Touiti, N. Rajhi, M. Bellali, N. Bram

**Affiliations:** Forensic Psychiatry Department, Razi Hospital, Manouba, Tunisia

## Abstract

**Introduction:**

Several studies have examined mortality in psychiatric settings, particularly among patients with schizophrenia showing that schizophrenia is associated with significantly higher mortality rates than those of the general population.

**Objectives:**

The aim of our work was to study mortality among patients with schizophrenia in the psychiatric wards of Razi Hospital (Manouba, Tunisia).

**Methods:**

A retrospective descriptive study was conducted over an 11-year period ; from January 2012 to December 2022 ; focusing on patients with schizophrenia who died during their stay at Razi Hospital. Data were collected from patients’ medical records. Sociodemographic and clinical characteristics as well as the medico-legal causes of their deaths were gathered.

**Results:**

A total of 37 patients were included. The mean age was 49.92 ± 14.13 years. Twenty-three patients (62.1 %) were male and 14 (22.7 %) were female. Analysis of socio-economic status showed that 24 patients (64.8 %) came from a low socio-economic background. Our study showed a history of psychiatric illness in first-degree relatives in 35.1 % of cases (N=13) and in second-degree relatives in 10.8 % (N=4) of cases. Fifteen patients (40.5 %) had a personal history of somatic illness. Seven (19.4%) had a criminal record. The different causes of deaths were pulmonary embolism (13.5 %,N=5), suicide (5.4 %, N=2), chronic obstructive pulmonary disease exacerbation (5.4 %,N=2), pulmonary oedema (2.7%, N=1), infectious pneumonia (2.7%, N=1), gastric perforation in (2.7%, N=1), status epilepticus (2.7% , N=1). The cause remained undetermined in 16.2 % (N=6) of cases.

**Conclusions:**

Schizophrenia remains associated with a markedly increased mortality risk. Our results emphasize the importance of integrated care approaches and preventive interventions to mitigate both natural and unnatural causes of death in this vulnerable population.

**Disclosure of Interest:**

None Declared

